# The Glutathione Derivative, GSH Monoethyl Ester, May Effectively Whiten Skin but GSH Does Not

**DOI:** 10.3390/ijms17050629

**Published:** 2016-04-27

**Authors:** Bo Young Chung, So Ra Choi, Ik Jun Moon, Chun Wook Park, Young-Hoon Kim, Sung Eun Chang

**Affiliations:** 1 Department of Dermatology, College of Medicine, Hallym University Kangnam Sacred Heart Hospital, Seoul 07441, Korea; victoryby@naver.com (B.Y.C.); dermap@hanmail.net (C.W.P.); 2 Department of Dermatology, Asan Medical Center, University of Ulsan College of Medicine, 88 Olympic-ro 43-gil, Songpa-gu, Seoul 05505, Korea; libesora@gmail.com (S.R.C.); ikjun.moon@gmail.com (I.J.M.); 3 Department of Pharmacology, University of Ulsan College of Medicine, 88 Olympic-ro 43-gil, Songpa-gu, Seoul 05505, Korea

**Keywords:** glutathione derivatives, glutathione monoethyl ester, melanogenesis, pheomelanin

## Abstract

Glutathione in its reduced form (GSH) is an antioxidant and also is involved in pheomelanin formation. Thus, it has been long believed that GSH has a skin whitening effect. However, its actual or direct effect is unproven. We evaluated the anti-melanogenic effects of GSH and its derivatives *in vitro*. We examined change of melanogenesis and its related proteins by GSH itself and its derivatives, including GSH monoethyl ester (GSH-MEE), GSH diethyl ester (GSH-DEE) and GSH monoisopropyl ester (GSH-MIPE) in Melan-A cells, Mel-Ab cells, and B16F10 cells. GSH and GSH-MEE did not display cytotoxic activity, but GSH-MIPE and GSH-DEE did. Intriguingly, GSH itself had no inhibitory effect on melanin production or intracellular tyrosinase activity. Rather, it was GSH-MEE and GSH-MIPE that profoundly reduced the amount of melanin and intracellular tyrosinase activity. Thus, GSH-MEE was selected as a suitable candidate skin-whitening agent and it did not alter melanogenesis-associated proteins such as microphthalmia-associated transcription factor (MITF), tyrosinase, tyrosinase-related protein (TRP)-1, and TRP-2, but it did increase the amount of suggested pheomelanin and suggested pheomelanin/eumelanin ratio. GSH-MEE was effective for anti-melanogenesis, whereas GSH itself was not. GSH-MEE could be developed as a safe and efficient agent for the treatment of hyperpigmentation skin disorders.

## 1. Introduction

Melasma and other skin hyperpigmentation disorders are often difficult to treat and often require multimodal approaches, including anti-melanogenesis (mostly anti-tyrosinase) and antioxidant strategies. Glutathione has long been considered and even used as an intravenous product for skin whitening and for the treatment of melasma, but its real or direct effect remains unproven. Because melasma is often refractory to various treatments, an anti-melasma oral formulation composed of cysteine (a component of GSH), Vitamin C, and tranexamic acid is also popular. A report [1] involving a small number of subjects in Thailand, and short-term follow up, showed that oral administration of glutathione resulted in lightening of skin color.

The skin is purposefully placed at the interface with the external environment where it has been developed to detect, incorporate and respond to various stressors including ultraviolet (UV) radiation [2]. Within the role of the skin as a stress organ, melanogenesis acts as a molecular sensor and transducer of noxious stimuli and as controller of local homeostasis [3]. l-tyrosine and l-dihydroxyphenylalanine (l-DOPA) not only serve as substrates and intermediates of melanogenesis, but are also known to be modulatory agents acting as inducers and positive regulators of melanogenesis as well as regulators of other cellular functions [4]. Although the primary function of melanin pigment and melanogenic apparatus is to protect the normal melanocytes from UV radiation and oxidative stress, at times, melanin can affect melanoma’s progression [5]. Melanogenesis is mostly affected by the activity of tyrosinase. The melanin pigment is produced from l-tyrosine that is converted to dopaquinone by the enzymatic activity of tyrosinase. This reaction proceeds spontaneously via dopachrome to the monomeric indolic precursors of eumelanin. Upon reaction with cysteine, dopaquinone forms 2- or 5-*S*-cysteinyldopa that produces benzothiazine precursors of pheomelanin polymer [3,6].

Glutathione, a tripeptide component of cysteine, glutamate and glycine, is a key antioxidant in the body and serves crucial roles in maintaining intracellular thiol status, and in detoxification of electrophilic metabolites as well as in xenobiotics. Although glutathione is present in its both reduced (GSH) and oxidized (GSSG) states, the majority of it found in the body is in the reduced form. GSH conducts antioxidant activity by scavenging free-radicals during reductive detoxification of hydrogen peroxide and lipid peroxide [7]. In addition to its antioxidant effect by removing free radicals and peroxides that assist tyrosinase activation and melanin production, GSH is known to be essential for pheomelanin formation because it mediates the mechanism for switching from eumelanin to pheomelanin production, which may provide another powerful strategy for skin whitening. Another proposed mechanism of GSH action involves the direct suppression of the tyrosinase activity when GSH binds to the copper-containing active site of tyrosinase [8].

Although intra-melanocytic GSH has been known to affect the regulation of melanogenesis, the effect of exogenous GSH is unclear. It is generally believed that GSH itself is not transported across the cell membrane. Moreover, the presence of GSH carriers for melanosomal transport has not been reported, nor does it seem likely that simple diffusion or a membrane channel could enable GSH entry into melanosomes.

Therefore, we hypothesized that the ester forms of GSH derivatives could have de-pigmenting efficacy superior to GSH, because the ester form of GSH derivatives could be delivered to intracellular spaces. In this study, we examined the anti-melanogenic efficacy of GSH and its derivatives, and sought to elucidate the underlying cellular mechanisms.

## 2. Results

### 2.1. GSH and GSH Monoethyl Ester (GSH-MEE) Did Not Affect Cellular Viability

**Figure 1:**
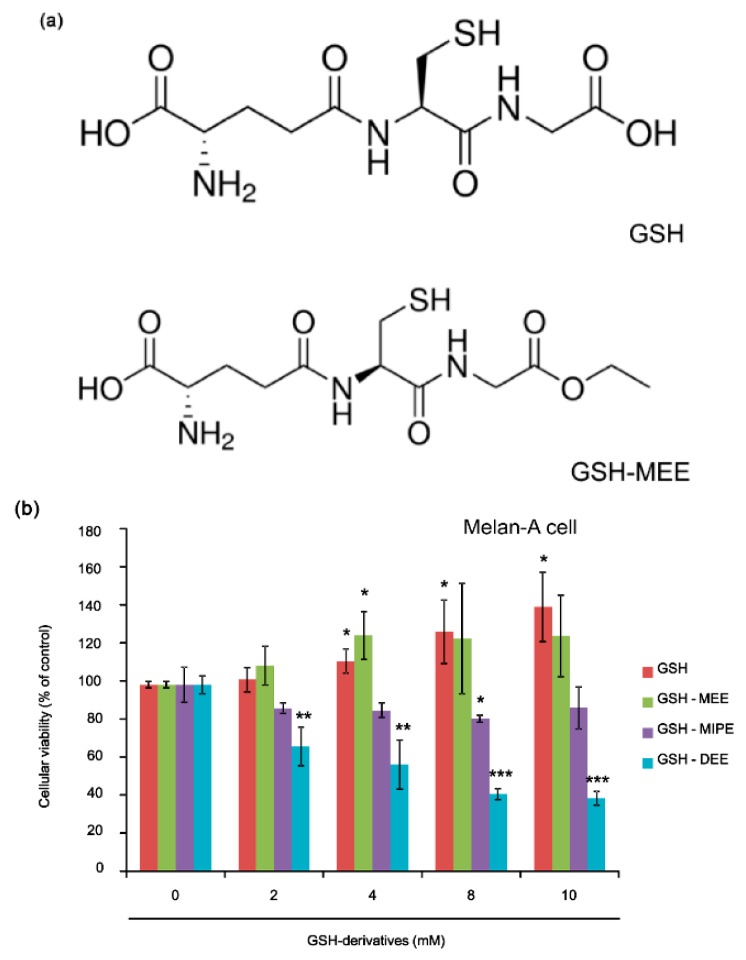
(**a**) Chemical structures of reduced glutathione (GSH) and GSH monoethyl ester (GSH-MEE); (**b**) Effect of GSH derivatives on cellular viability. GSH and GSH-MEE had no effect on cellular viability, but GSH-MIPE and GSH-DEE showed toxicity in Melan-A cells. Data are shown as the mean ± SD of three replicates, and * *p* < 0.05, ** *p* < 0.01, and *** *p* < 0.001 compared to untreated controls. GSH, reduced glutathione; GSH-MEE, GSH monoethyl ester; GSH-MIPE, GSH monoisopropyl ester; GSH-DEE, GSH diethyl ester; SD, standard deviation.

GSH and GSH-MEE (Figure 1a) did not affect cellular viability in Melan-A cells (Figure 1b). GSH monoisopropyl ester (GSH-MIPE) had slight cellular toxicity (80% cell viability of control at 8 mM, Figure 1b) and GSH diethyl ester (GSH-DEE) had a profound cellular toxicity in Melan-A cells, even at concentrations as low as 2 mM (Figure 1b). For this reason, GSH-DEE was not used in subsequent experiments.

### 2.2. Whereas GSH Itself Did Not Suppress Melanogenesis, Its Derivative GSH-MEE Did Reduce Melanogenesis

**Figure 2:**
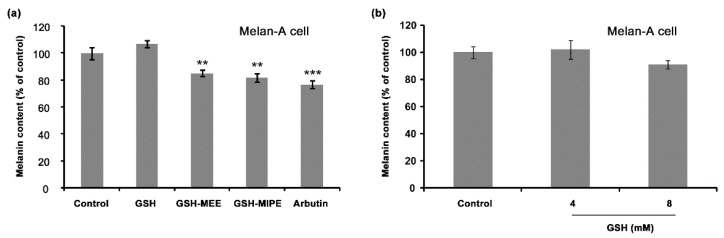

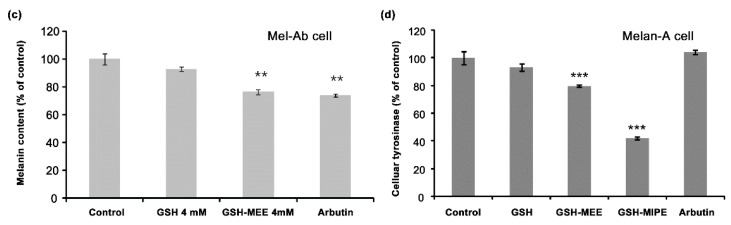
(**a**) Effect of GSH derivatives on melanin content in Melan-A cells. GSH exhibited no inhibitory effect on melanin production, but GSH-MEE and GSH-MIPE decreased the melanin synthesis in Melan-A cells. Arbutin (100 µg/mL) was treated to serve as a positive control. The melanin content was calculated by normalizing the melanin content to total cellular protein and reported as a percentage of control. Data are shown as the mean ± SD of three replicates, and ** *p* < 0.01, and *** *p* < 0.001. GSH, reduced glutathione; GSH-MEE, GSH monoethyl ester; GSH-MIPE, GSH monoisopropyl ester; SD, standard deviation; (**b**) The effect of GSH on melanin production in Melan-A cells. GSH had no inhibitory effect on melanin production in Melan-A cells. Each sample was quantified with the same amount of protein and reported as a percentage of control. Data are shown as the mean ± SD of three replicates; (**c**) Effect of GSH and GSH-MEE on melanin content in Mel-Ab cells GSH had no inhibitory effect on melanin production, but GSH-MEE decreased the synthesis of melanin significantly in Mel-Ab cells. Arbutin (100 µg/mL) was treated as a positive control. The melanin content was calculated by normalizing the melanin content to total cellular protein and reported it as a percentage of control. Data are shown as the mean ± SD of three replicates, and ** *p* < 0.01; (**d**) the effect of GSH derivatives on intracellular tyrosinase activity in Melan-A cells. GSH had no significant effect on intracellular tyrosinase activity, but GSH-MEE and GSH-MIPE decreased the activity of intracellular tyrosinase in Melan-A cells. Arbutin (100 µg/mL) was treated as a positive control. Each sample was quantified with the same amount of protein and reported as a percentage of control. Data are shown as the mean ± SD of three replicates, and *** *p* < 0.001.

We then determined the effect of GSH, GSH-MEE, and GSH-MIPE on melanogenesis. Surprisingly, GSH had no inhibitory effect on melanogenesis: the melanin content did not change at any concentration tested (Figure 2a,b). However, GSH-MEE and GSH-MIPE decreased melanin production in Melan-A cells (Figure 2a). The decrease of melanin content by GSH-MEE was reproduced in another melanocyte-derived cell line, Mel-Ab (Figure 2c). We next examined the effects of GSH, GSH-MEE, and GSH-MIPE on tyrosinase activity. In Melan-A cells, as expected, GSH did not affect intracellular activity of tyrosinase, but GSH-MEE and GSH-MIPE inhibited the activity of intracellular tyrosinase (Figure 2d). Although both GSH-MEE and GSH-MIPE showed evident antimelanogenic effects, GSH-MEE was considered a more promising candidate de-pigmenting agent because it did not compromise cellular viability.

**Figure 3:**
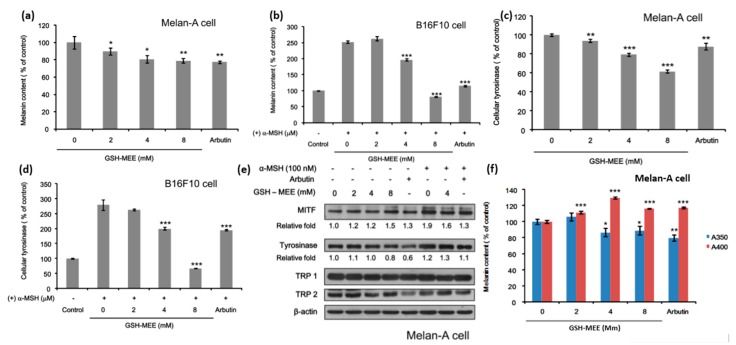
(**a**) Effect of GSH-MEE on melanin production in Melan-A cells. GSH-MEE decreased the production of melanin in a dose-dependent manner in Melan-A cells. Arbutin (100 µg/mL) was treated as a positive control. The melanin content was calculated by normalizing the melanin content to total cellular protein and reported as a percentage of control. Data are shown as the mean ± SD of three replicates, and * *p* < 0.05, ** *p* < 0.01. GSH-MEE, reduced glutathione monoethyl ester; SD, standard deviation; (**b**) Effect of GSH-MEE on melanin content in B16F10 cells. GSH-MEE decreased the amount of melanin produced by B16F10 cells. Arbutin (100 µg/mL) was treated as a positive control. The melanin content was calculated by normalizing the melanin contents to total cellular protein and reported as a percentage of control. Data are shown as the mean ± SD of three replicates, and *** *p* < 0.001. GSH-MEE, reduced glutathione monoethyl ester; α-MSH, α-melanocyte-stimulating hormone; SD, standard deviation; (**c**) Effect of GSH-MEE on intracellular tyrosinase activity in Melan-A cells. GSH-MEE decreased the activity of intracellular tyrosinase in a dose-dependent manner in Melan-A cells. Arbutin (100 µg/mL) was treated as a positive control. Each sample was quantified with the same amount of protein and reported as a percentage of control. Data are shown as the mean ± SD of three replicates, and ** *p* < 0.01, and *** *p* < 0.001. GSH-MEE, reduced glutathione monoethyl ester; SD, standard deviation; (**d**) Effect of GSH-MEE on intracellular tyrosinase activity in B16F10 cells. GSH-MEE decreased the activity of intracellular tyrosinase in B16F10 cells. Arbutin (100 µg/mL) was treated as a positive control. Each sample was quantified with the same amount of protein and reported as a percentage of control. Data are shown as the mean ± SD of three replicates, and *** *p* < 0.001. GSH-MEE, reduced glutathione monoethyl ester; α-MSH, α-melanocyte-stimulating hormone; SD, standard deviation; (**e**) Effect of GSH-MEE on the expression of melanogenesis-related proteins. When Melan-A cells were treated with GSH-MEE, the expression levels of MITF, tyrosinase, TRP-1, and TRP-2 did not change significantly. Arbutin (100 µg/mL) was treated as a positive control, and β‑actin expression was used as the loading control. These results represents three independent experiments. GSH-MEE, reduced glutathione monoethyl ester; MITF, microphthalmia-associated transcription factor; TRP-1, tyrosinase-related protein-1; TRP-2, tyrosinase-related protein-2; (**f**) Effect of GSH-MEE on suggested eumelanin and pheomelanin production. When Melan-A cells were treated with GSH-MEE, the synthesis of melanin with absorbance at 400 nm (A400, suggestive of eumelanin) increased, whereas the production of melanin with absorbance at 350 (A350, suggestive of pheomelanin) decreased. Arbutin (100 µg/mL) was treated as a positive control. The melanin content was calculated by normalizing the melanin contents to total cellular proteins and reported as a percentage of control. Data are shown as the mean ± SD of three replicates, and * *p* < 0.05, ** *p* < 0.01, and *** *p* < 0.001. GSH-MEE, reduced glutathione monoethyl ester; A400, absorbance at 400 nm; A350, absorbance at 350 nm; SD, standard deviation.

Therefore, further experiments were focused on GSH-MEE. To evalute how the concentration of GSH-MEE affects melanongenesis, cells were treated with 2–8 mM GSH-MEE. Two days after, the melanin content was measured. The results revealed the negative regulatory effect of GSH-MEE on melanin synthesis in both Melan-A cells and B16F10 cells (Figure 3a,b). The intracellular tyrosinase activity was also suppressed in both cells (Figure 3c,d), and the responses of melanogenesis and tyrosinase activity to various doses of GSH-MEE were in accordance with each other. However, GSH-MEE did not reduce the protein levels of tyrosinase, microphthalmia-associated transcription factor (MITF), tyrosinase-related protein (TRP)-1, and TRP-2 significantly at the test-doses above (Figure 3d). These data suggest that GSH-MEE regulates intracellular activity of tyrosinase and subsequently downregulates melanin synthesis.

### 2.3. GSH-MEE Suppresses Levels of the Melanin with Absorbance at 350 nm (A350) Production, but Induces the Melanin with Absorbance at 400 nm (A400) Production in Melan-A Cells

Finally, we sought to determine whether the GSH-MEE-mediated decrease in melanin synthesis results from its regulation of suggested pheomelanin and eumelanin production. Melan-A cells were incubated with GSH-MEE for 48 h, and then the suggested eumelanin and pheomelanin content was measured using spectrophotometric analysis. GSH-MEE increased the melanin with A400 production and decreased the melanin with A350. (Figure 3e). The A350 values are referred to as spectrophotometric eumelanin and the A400 values are referred to as spectrophotometric pheomelanin. Taken together, these results indicate that GSH-MEE may have a de-pigmenting effect by inhibiting tyrosinase activity, resulting in reduction of suggested eumelanin synthesis and increase of suggested pheomelanin synthesis.

## 3. Discussion

The majority of glutathione is present in its reduced form, GSH, which is a well-known antioxidant. Accordingly, GSH is expected to repress melanin production by redox mechanisms. Exhaustion of GSH in the skin by UV radiation might induce the accumulation of hydrogen peroxide as a result of insufficient detoxification. Subsequently, increased melanin synthesis could be further promoted by hydrogen peroxide or other reactive oxygen species [9,10,11]. On the other hand, for more than 30 years, GSH has been known to be a regulatory molecule in mammalian melanogenesis, that is, an endogenous reductant diverting dopaquinone to pheomelanin pathway ultimately leading to skin whitening. In addition, it was proposed that GSH affects melanogenesis by inhibiting tyrosinase activity [12,13,14,15]. Tyrosinase plays the main enzymatic role in melanogenesis, catalyzing the conversion of l-tyrosine into l-dopaquinone. It was reported thiol groups directly inhibit tyrosinase by acting on copper at the active site of the enzyme [16]. For this reason, the skin whitening effect of exogenous GSH has been assumed for long, even though the effect of an intravenous product for the treatment of melasma and other hyperpigmentary disorders was unclear and unproven. Thus, the data regarding the effect of GSH on pigmentation is inconclusive [1].

**Figure 4:**
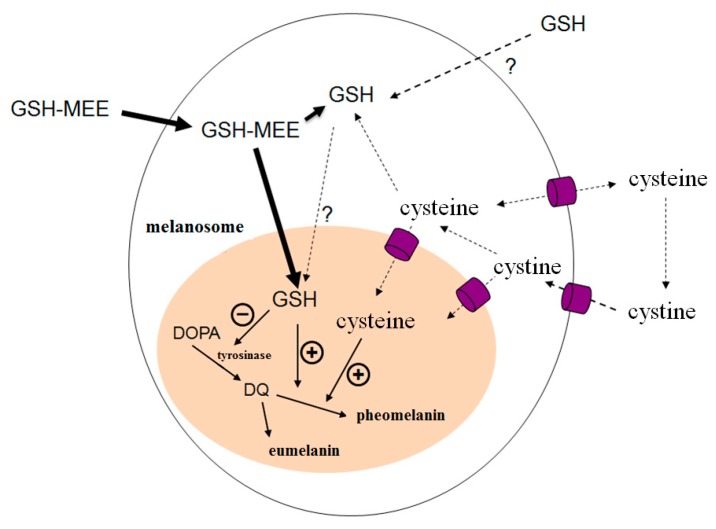
Possible effects of GSH-MEE, GSH, and cysteine on melanogenesis. GSH synthesize intracellularly and exported to extracellular space. Cellular uptake of GSH itself is not probable. GSH-MEE cross the biological membranes by simple diffusion and metabolize intracellularly to generate GSH. Cysteine is readily transported across cell membranes but potentially toxic at high concentrations due to generation of reactive oxygen species and depletion of pyridoxal phosphate. GSH-MEE (but not GSH itself) could suppress the melanin production and tyrosinase activity and raise the suggested pheomelanin/eumelanin ratio. black arrows, melanin synthesis process flow; dotted black arrows, transport process flows of GSH, cysteine, and cysteine, bold black arrows, transport process flow of GSH-MEE; question marks, not yet confirmed; +, positive effects (activation); −, negative effects (suppression); purple shape, membrane channel. GSH, reduced glutathione; GSH-MEE, GSH monoethyl ester; DOPA, dihydroxyphenylalanine; DQ, dopaquinone.

Also, if GSH had a direct regulatory effect on melanogenesis, at the least, it would be required that GSH be transported into melanosomes. Carrier-mediated lysosomal membrane transport systems remain for cysteine [17]. However, GSH is not transported into melanosomes, nor is GSH likely to be capable of enter melanosomes by simple diffusion or through a membrane channel. (Figure 4) [18].

Therefore, we hypothesized that the ester form of a GSH derivative would be more effective at entering melanosomes because the GSH ester form is more lipophilic (Figure 4). In our study, we determined the effects of GSH and GSH derivatives on melanin synthesis, and evaluated the cellular mechanism of the regulation of melanogenesis *in vitro*.

Intriguingly, we found that GSH did neither suppress the enzymatic activity of tyrosinase, nor reduce melanin synthesis. However, its derivatives, GSH-MEE and GSH-MIPE did reduce melanin synthesis. These findings are consistent with our null hypothesis that the esterified derivatives of GSH would show superior efficacy because of the feasibility of intracellular and intra-melanosomal delivery [19,20]. In our study, among the esterified derivatives of GSH, the most suitable candidate whitening agent was GSH-MEE. Furthermore, GSH-MEE has a lipophilic nature that makes it better able to penetrate skin as a topical agent.

GSH-MEE demonstrated itself to be an effective compound for inhibiting cellular melanin synthesis, and was also not cytotoxic, unlike the other esterified derivatives of GSH, GSH-MIPE and GSH-DEE. We further evaluated the regulation mechanism of the anti-melanogenic effect caused by GSH-MEE. It did not significantly decrease the levels of melanogenesis-associated proteins such as MITF, tyrosinase, TRP-1, and TRP-2, but it increased the amount of suggested pheomelanin and the ratio of suggested pheomelanin/eumelanin. The biosynthesis of melanin takes place through a complex but orderly sequence of enzymatic and chemical reactions. The melanins produced are conventionally classified into two major types: eumelanins (brown or black) and sulfur-containing pheomelanins (red or yellow) [21]. Within the melanocytes, sulfhydryl compounds such as GSH and its precursor cysteine, are actively involved in the melanogenic process of making pheomelanin [6]. Besides their essential roles in pigmentation, the intracellular concentrations of GSH and cysteine are also a crucial regulator of the switch of eumelanin to pheomelanin [22]. Decreased tyrosinase activity is also associated with a more marked shift to pheomelanin synthesis, as it is considered to be the default pathway [23].

A recent study showed anti-melanogenic effects of exogenous GSH in melanoma cells [24]. We speculated that the discrepancy might be related with the extracellular metabolism of GSH. γ-glutamyltranspeptidase (GGT), an ectoenzyme involved in the degradation of GSH derivatives exported from cytosol cleaves the γ-glutamyl peptide bond of GSH to generate cysteinylglycine and transfers the γ-glutamyl group to another amino acid including cysteine [20].

The first limitation of this study to be addressed was using the spectrophotometric analysis to measure concentrations of eumelanin and pheomelanin. The measurement of degraded products by high-performance liquid chromatography (HPLC) or the noninvasive method of electron paramagnetic resonance (EPR) are more analytical methods to measure concentrations of eumelanin and pheomelanin than the spectrophotometric analysis used in this study [25,26]. The second limitation of this study was the strict focus on mouse melanocytes lines and inconsistent use of cell lines. Therefore, further studies to determine the anti-melanogenic effect of GSH-MEE on other mouse melanocytes and human melanocytes will be needed.

## 4. Materials and Methods

### 4.1. Reagents

GSH and its derivatives (GSH-MEE, GSH-DEE, and GSH-MIPE) were acquired from Sigma-Aldrich (St. Louis, MO, USA) and Santa Cruz Biotechnology (Santa Cruz, CA, USA), respectively. Primary antibodies against mouse proteins were acquired from the following sources: anti-β-actin (Sigma-Aldrich), anti-MITF (NeoMarkers, Fremont, CA, USA), anti-tyrosinase (Santa Cruz, CA, USA), anti-TRP-1, and anti-TRP-2 (Abcam, Cambridge, UK).

### 4.2. Cells and Cell Culture

Melan-A mouse melanocytes were cultured in RPMI 1640 (Gibco, Carlsbad, CA, USA), containing Phorbol 12-myristate 13-acetate (PMA; 200 nM), 10% fetal bovine serum (FBS, Gibco), and 1% antibiotic-antimycotic 100× (Gibco). The Mel-Ab cell line is a mouse-derived spontaneously immortalized cell line of melanocytes [27]. Mel-Ab cells were contained in Dulbecco’s modified Eagle’s medium (DMEM, Welgene, Deagu, Korea) mixed with 10% FBS, 100 nM 12-*O*-tetradecanoylphorbol-13-acetate (TPA), 1 nM cholera toxin (CT), 50 µg/mL streptomycin, and 50 U/mL penicillin. The B16F10 mouse melanoma cells were obtained from the Korean Cell Line Bank (Seoul, Korea). The B16F10 mouse melanoma cells were incubated in DMEM (Welgene), supplemented with 10% FBS (Gibco) and 1% (*v*/*v*) penicillin-streptomycin (Gibco). All cells were grown at 37 °C in a humidified atmosphere containing 5% CO_2_.

### 4.3. Cellular Viability Analysis

Following treatment with each of the GSH and its derivatives for two days, cellular viability was determined using EZ-Cytox cell growth assay kit (ITSBIO, Seoul, Korea). Briefly, cells were seeded in triplicate in 96-well plates at a density 1 × 10^3^ cells/well. EZ-Cytox was added at 10 µL/well and the plates were incubated at 37 °C for 30 min to 2 h. Absorbance was measured at 450 nm using an enzyme immunosorbent assay (ELISA) reader (Molecular Devices Co., Sunnyvale, CA, USA) with the reference wavelength 600–650 nm.

### 4.4. Total Melanin Content Assay and Spectrophotometric Determination of Eumelanin and Pheomelanin

Total melanin content was measured as described previously [28]. Briefly, Melan-A cells were treated with each of the GSH and its derivatives in culture medium containing PMA (200 nM) for 48 h. B16F10 cells were incubated at a density of 1 × 10^5^ cells in 6-well plates overnight. α-melanocyte-stimulating hormone (α-MSH, 200 nM) was then added and cells were treated with increasing concentrations of the GSH and its derivatives in phenol red-free DMEM for 3 days. Cells were re-suspended in 600 µL of 1 g/L NaOH at 100 °C for 30 min and centrifuged at 13,000 rpm for 5 min. The optical densities (OD) of the supernatants were measured using an ELISA reader at 405 nm. The melanin content was calculated by normalizing the melanin content to total cellular protein content, as measured by a Micro BCA assay kit (Pierce Biotechnology, Rockford, IL, USA).

In separate experiments, contents of eumelanin and pheomelanin candidates were measured using spectrophotometric assay methods specific for the respective melanin type [29,30,31]. For the spectrophotometric measurement of eumelanin, 2.4 × 10^7^ cells were heated for 20 h at 130 °C in 500 µL hydriodic acid with 30 µL H_2_O_2_, and insoluble eumelanin was solubilized by heating for 30 min at 100 °C in 1 mol·L^−1^ NaOH in the existence of H_2_O_2_, and analysed for A350 [30]. The A350 values are suggested as eumelanin. For the spectrophotometric assay of pheomelanin, 1.6 × 10^7^ cells were isolated with 0.1 mol·L^−1^ sodium phosphate buffer, pH 10.5. Supernatants were analyzed for A400. These values are suggested as pheomelanin.

### 4.5. Intracellular Tyrosinase Activity Assay

Tyrosinase activity assay was performed as described previously [31,32]. Briefly, Melan-A cells and B16F10 cells were seeded in 6-well plates and incubated with GSH derivatives for 2 or 3 days. The cells were washed with ice-cold PBS and lysed with phosphate buffer (pH 6.8) containing 1% Triton X-100. They were then discomposed by freezing and thawing, and lysates were clarified by centrifugation at 15,000 rpm for 10 min. After measuring the protein levels of the lysate and adjusting the protein concentrations with lysis buffer, 90 mL of each lysate containing equal amounts of protein was added in each well of a 96-well plate, and 10 mL of 10 mM l-dihydroxyphenylalanine (l-DOPA) was then placed in each well. The control wells were composed of 90 mL of lysis buffer and 10 mL of 10 mM l-DOPA. Following incubation at 37 °C, absorbance at 475 nm was analysed every 10 min for at least 1 h using a microplate reader.

### 4.6. Western Blot Analysis

Total proteins were isolated using PRO-PREP extraction solution (Intron, Daejeon, Korea). Protein concentrations were evaluated using the BCA Protein Assay Reagent (Pierce). Equally loaded proteins from each sample were resolved in SDS-PAGE gels (Invitrogen, Merelbeke, Belgium) and transferred to nitrocellulose membranes (Whatman™, GE Healthcare Life Sciences, Buckinghamshire, UK), which were blocked for 1 h at room temperature in tris buffered saline with Tween 20 (TBS-T) and 5% nonfat dry milk. The membrane was then incubated over-night at a temperature of 4 °C with the primary antibody diluted in 5% nonfat dry milk, or 5% bovine serum albumin (BSA) mixed in TBS-T. The membranes were then washed, and incubated for another 1 h at room temperature with horseradish peroxidase-conjugated secondary antibody (Cell Signaling Technology). After additional washing with TBS-T, protein bands were visualized by the use of a SuperSignal West Pico Trial kit (Thermo Scientific, Rockford, IL, USA). Primary antibodies against the following proteins were used: β-actin, MITF, tyrosinase, TRP-1, and TRP-2.

### 4.7. Statistical Analysis

The statistical significance of any differences between groups was determined by the analysis of variance (ANOVA), followed by the Dunnett’s test or Tukey’s test. *p* Values <0.05 were considered to be significant statistically.

## 5. Conclusions

In our study, esterified GSH derivative, particularly GSH-MEE (but not GSH itself), was effective without affecting cellular viability for reducing melanin and tyrosinase activity and raising the suggested pheomelanin/eumelanin ratio. Taken together, this novel esterified GSH derivative could be developed as a safe and efficient agent for the treatment of hyperpigmentary skin disorders.

## Abbreviations

GSH	Reduced glutathione
UV	Ultraviolet
l-DOPA	l-dihydroxyphenylalanine
GSSG	Oxidized glutathione
GSH-MEE	GSH monoethyl ester
GSH-DEE	GSH diethyl ester
GSH-MIPE	GSH monoisopropyl ester
MITF	Microphthalmia-associated transcription factor
TRP-1	Tyrosinase-related protein-1
TRP-2	Tyrosinase-related protein-2
PMA	Phorbol 12-myristate 13-acetate
FBS	Fetal bovine serum
DMEM	Dulbecco’s modified Eagle’s medium
TPA	12-*O*-tetradecanoylphorbol-13-acetate
CT	Cholera toxin
ELISA	Enzyme immunosorbent assay
α-MSH	α-melanocyte-stimulating hormone
OD	Optical densities
A350	Absorbance at 350 nm
A400	Absorbance at 400 nm
TBS-T	Tris buffered saline with Tween 20
BSA	Bovine serum albumin
ANOVA	Analysis of variance
GGT	γ-glutamyltranspeptidase
HPLC	High-performance liquid chromatography
EPR	Electron paramagnetic resonance
